# Proactive and Reactive Stopping When Distracted: An Attentional Account

**DOI:** 10.1037/a0036542

**Published:** 2014-05-19

**Authors:** Frederick Verbruggen, Tobias Stevens, Christopher D. Chambers

**Affiliations:** 1 School of Psychology, University of Exeter, Exeter, United Kingdom; 2 School of Psychology, Cardiff University, Cardiff, Wales, United Kingdom

**Keywords:** perceptual distraction, response inhibition, proactive control, response strategies, signal monitoring

## Abstract

Performance in response inhibition paradigms is typically attributed to inhibitory control. Here we examined the idea that stopping may largely depend on the outcome of a sensory detection process. Subjects performed a speeded go task, but they were instructed to withhold their response when a visual stop signal was presented. The stop signal could occur in the center of the screen or in the periphery. On half of the trials, perceptual distractors were presented throughout the trial. We found that these perceptual distractors impaired stopping, especially when stop signals could occur in the periphery. Furthermore, the effect of the distractors on going was smallest in the central stop-signal condition, medium in a condition in which no signals could occur, and largest in the condition in which stop signals could occur in the periphery. The results show that an important component of stopping is finding a balance between ignoring irrelevant information in the environment and monitoring for the occurrence of occasional stop signals. These findings highlight the importance of sensory detection processes when stopping and could shed new light on a range of phenomena and findings in the response inhibition literature.

Goal-directed behavior requires an executive control system that allows us to ignore irrelevant information, replace responses, and adjust processing strategies in demanding situations. Here, we investigated how these control functions interact in a stop-signal task, which is a popular tool to examine the behavioral and neural correlates of inhibition in healthy and clinical populations (Verbruggen & Logan, 2008). Researchers typically attribute performance in this task, and in related paradigms, to the effectiveness of a single inhibitory control function. But by referring to a general construct such as inhibition, we cannot adequately explain stop-signal performance. We have recently proposed a theoretical framework which proposes that various forms of action control depend on three basic cognitive processes: signal detection, action selection, and action execution. These processes are modulated via correction- or evaluation mechanisms, preparation, task rules maintained in memory, and learning (Verbruggen, McLaren, & Chambers, 2014). The aim of this framework is to eliminate the control homunculi from theories of action control.

In the present study, we tested part of this framework by demonstrating that stopping critically depends on signal detection. In a stop-signal task, subjects respond to a go stimulus on no-signal trials. On a random selection of the trials (stop-signal trials), a stop signal is presented after a variable delay (stop-signal delay; SSD), which instructs subjects to withhold their response to the go stimulus. The first index of “inhibitory” control is the probability of responding on stop-signal trials, *p*(respond|signal) (Logan & Cowan, 1984). The second index of inhibitory control is an estimate of the covert latency of the stop process, stop-signal reaction time (SSRT). *P*(respond|signal) and SSRT are both measures of *reactive* control on stop-signal trials. The third index is go reaction time (RT) on no-signal trials. RT is typically longer in blocks in which stop signals can occur than in blocks in which no signals occur. This RT difference has been interpreted as a measure of *proactive* control: people increase response thresholds and generally suppress motor output in situations in which stop signals can occur, compared with situations in which they can always respond (e.g., Cai, Oldenkamp, & Aron, 2011; Jahfari et al., 2012; Verbruggen & Logan, 2009). Thus, there are three main dependent variables in a stop-signal task, and most researchers use them to study the inhibition of motor output. However, our theoretical framework states that noninhibitory processes also play a critical role in stopping responses. The first step in successfully cancelling a response is nearly always detecting the stop signal (e.g., a traffic light turning red or noticing an obstacle on the road). Computational work even suggests that most of SSRT is occupied by afferent or sensory processes (Boucher, Palmeri, Logan, & Schall, 2007; Salinas & Stanford, 2013). Thus, despite the fact that the contribution of nonmotor processes is largely neglected in the literature, it appears that stopping on signal trials largely depends on the outcome of sensory processes. Because a failure to detect the signal quickly could have important negative consequences, people may also adjust attentional settings in advance when they expect a stop signal (e.g., preparing oneself to detect the red light or directing spatial attention to the location of possible obstacles). In other words, proactive control may also involve adjusting perceptual processes.

We used a perceptual load manipulation in a stop-signal task to demonstrate that perceptual processes are a key component of both reactive and proactive control in response inhibition paradigms. Subjects responded to centrally presented words on no-signal trials (see Figure 1). In some blocks, a stop signal was presented on a random 33% of the trials. There were three types of blocks: *central-signal* blocks, in which a visual stop signal could occur in the center of the screen, *noncentral-signal* blocks, in which a visual stop signal could occur in the periphery, and *no-signal* blocks, in which no stop signals could occur. On a random 50% of the no-signal and signal trials, visual distractors were presented. Based on previous work, we assumed that subjects would focus their attention on the center of the screen (i.e., narrow the “attentional spotlight”) when distractors appeared.[Fig-anchor fig1]

To examine the role of stop-signal detection, we estimated SSRT as a function of distractor presentation and stop-signal type.^1^ Narrowing the focus of attention on distractor trials would make detection of stop signals in the periphery harder. Consequently, our attentional account of reactive stopping predicts that the effect of distractors on SSRTs will be larger in noncentral-signal blocks than in central-signal blocks.

To examine proactive attentional control adjustments, we compared RT on no-signal trials in the three signal conditions. Our attentional account predicts that subjects would normally direct their attention to the location of the stop signal. But in noncentral-signal blocks, this creates a trade-off between stop-signal detection and interference control: On the one hand, subjects try to widen the attentional focus to detect stop signals in the periphery; on the other hand, they try to narrow their focus to avoid processing of distractors. These opposing demands are expected to result in a larger distractor effect on no-signal performance in noncentral-signal blocks than in no-signal blocks without the opposing attentional demands. By contrast, the proactive attentional adjustments could result in a smaller distractor effect in central-signal blocks than in no-signal blocks. In the no-signal blocks, a narrow focus of attention is not strictly required, especially because the stimuli of the primary task are presented above and below the center of the screen. In the central stop-signal blocks, subjects are strongly encouraged by the task demands to focus on the center of the screen. Consequently, the distractor effect would be smaller in central-signal blocks than in no-signal blocks.

## Experiment

### Method

#### Subjects

Twenty-four students from the University of Exeter participated for monetary compensation (£6). Two subjects were replaced because the percentage of correct go trials was ≤75%. The data with these subjects included are available online as supplemental material. The target sample size and exclusion criteria were decided in advance of data collection.

#### Apparatus and stimuli

**Figure 1 fig1:**
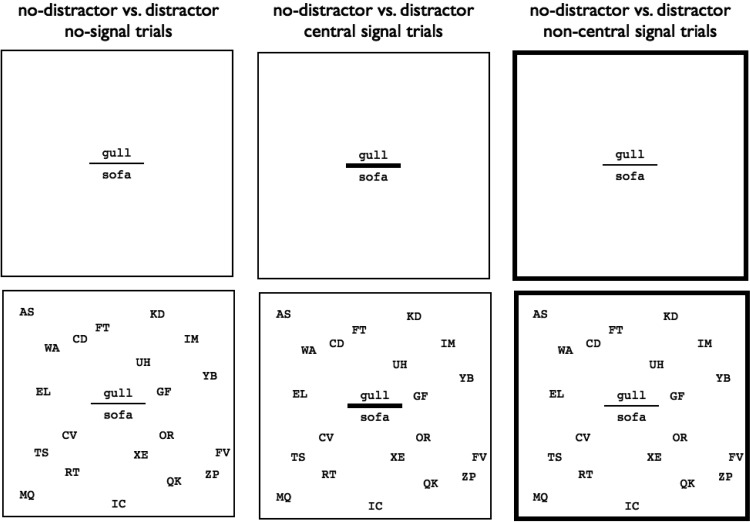
Examples of the six possible trials types (see Method section for further details). On no-signal trials, half of the subjects responded to the location of the natural object; the other half to the location of the man-made object. On distractor trials, random two-letter strings appeared at random locations every 100 ms. On signal trials in the central signal condition, the central line turned bold after a variable delay (SSD); on signal trials in the noncentral condition, the large square turned bold after the SSD. On such signal trials, subjects tried to withhold a response. Stop signals always occurred after the presentation of the go stimulus and the distractors. For display purposes, foreground and background colors are switched (i.e., in the experiment, white stimuli appeared against a black background). A short Quicktime movie with an example of a trial sequence is deposited on the Open Research Exeter data repository (http://hdl.handle.net/10871/13401). Please note that this is an example of a trial in the pilot study; consequently, there are only 15 distractors).

The experiment was run on a PC using Psychtoolbox (Brainard, 1997). The stimuli were presented on a 17-in CRT monitor. The go stimuli were 54 four-letter words (available online as supplementary material). For every subject, we created nine subsets of six words (one subset per block).^2^ On each trial, two words were presented in white lowercase font (Courier 16 point; visual angle: 1.5° × 0.4°) on a black background (see Figure 1). One word referred to a natural object, and the other to a man-made object. The words appeared on either side (distance: 0.6°) of a central white line (1.8°), inside a white rectangle (10.5° × 10.5°). Half of the subjects responded to the location of the natural object; the other half to the location of the man-made object. They responded by pressing the Up or Down arrow keys of a keyboard using the right index finger. On distractor trials, 20 randomly generated two-letter uppercase strings (Courier 16; 0.8° × 0.5°) were presented at random locations within the square. To avoid overlap between the distractors and words, the center of the distractors was outside a smaller central region (3.1° × 3.1°).^3^

An EyeLink 1000 Desktop Mount camera system (SR Research, Ottawa, Canada), calibrated before each block, tracked the gaze position of the right eye during the whole block.

#### Procedure

All trials started with the presentation of the square and the central horizontal line. After 250 ms, the two words appeared. On half of the trials in each block (distractor trials), 20 distractors also appeared. Every 100 ms, 20 new distractors appeared at new random locations to ensure a perceptual load during the whole trial; this was required because the delay between the go stimulus and the stop signal varied (see below). After 1,500 ms, the words and distractors were replaced by a feedback message (on no-signal trials: *correct*, *incorrect*, or *not quick enough* in case they did not respond before the end of the trial; on signal trials: *correct stop* or *failed stop*), which remained on the screen for 500 ms. The feedback was presented to encourage fast and accurate responding. The next trial started immediately after the feedback.

In the central- and noncentral-signal blocks, a stop signal was presented on 33% of the trials (stop-signal trials). In the central-signal blocks, the central line turned bold (1 to 3 pixels) on signal trials; in the noncentral-signal blocks, the outline of the surrounding square turned bold (1 to 3 pixels). The line(s) turned bold after a variable stop-signal delay (SSD). SSD was initially set at 500 ms, and continuously adjusted according to a tracking procedure to obtain a probability of stopping of .50: SSD decreased by 50 ms when a subject responded on a stop-signal trial, but increased by 50 ms when they successfully stopped. We used separate tracking procedures for central- and noncentral-signal blocks and for trials with and without distractors.

Each condition consisted of three blocks of 108 trials (total number of trials per condition: 324), resulting in nine blocks overall. Order of the blocks (no-signal, central-signal, noncentral-signal) was counterbalanced across subjects (e.g., NS-CS-NCS-NS-CS-NCS-NS-CS-NCS). At the beginning of each block, a message on the screen informed subjects whether central or noncentral stop signals could occur. At the end of each block, we presented as feedback to the subject their mean RT on no-signal trials, number of no-signal errors and missed no-signal responses, and percentage of failed stops.

#### Analyses

All data processing and analyses were completed using R (R Development Core Team, 2014). Proactive response-strategy adjustments could result in a higher percentage of omitted responses as well as higher accuracy (Verbruggen & Logan, 2009), so we distinguished between the proportion of correct no-signal trials and the proportion of missed no-signal trials. SSRTs were estimated using the session-wide integration method (Verbruggen, Chambers, & Logan, 2013). The distractor effect refers to performance on distractor trials minus performance on no-distractor trials. See online supplemental material for exploratory analyses of the eye data.

All data files and R scripts used for the analyses of the pilot study and the experiment reported here are deposited on the Open Research Exeter data repository (http://hdl.handle.net/10871/13401).

### Results and Discussion

An overview of the data and analyses appears in Tables 1 and 2. The stopping latencies support the “attentional” account of reactive control: SSRTs were longer on distractor trials (493 ms) than on no-distractor trials (348 ms), *p* < .001. Importantly, the distractor effect on SSRTs was much larger in noncentral-signal blocks (253 ms) than in central-signal blocks (37 ms). This interaction was reliable (*p* < .001; Table 2). The attentional account is further supported by the exploratory analyses of the eye data: the frequency of eye movements increased in the noncentral-signal condition (available online as supplemental material). Compared with previous stop-signal studies, SSRTs were much longer on distractor trials in the noncentral-signal condition. It is possible that on a proportion of the stop-signal trials with distractors, subjects responded because they did not detect the stop signal in time. This could have inflated SSRT estimates (Band, van der Molen, & Logan, 2003). Thus, the absolute value of SSRTs should be interpreted with caution. But even if SSRT is inflated, the difference between conditions still points to a stopping deficit caused by perceptual factors because responding on a stop-signal trial is generally considered as one of the main indices of control (Logan & Cowan, 1984). [Table-anchor tbl1][Table-anchor tbl2]

Next, we analyzed no-signal RTs. On average, distractors slowed responding on no-signal trials by 70 ms, and RTs were longer in central (975 ms) and noncentral signal blocks (987 ms) than in no-signal blocks (754 ms). These main effects were statistically significant (see Table 2). Two-tailed *t* tests revealed that the difference between the no-signal blocks and the central- and noncentral-signal blocks were significant, *t*(23) = 6.87, *p* < .001, Cohen’s *d* = 1.4, and *t*(23) = 7.58, *p* < .001, Cohen’s *d* = 1.5, respectively. The difference between the two signal conditions was not significant, *t*(23) = 1.16, *p* = .26, Cohen’s = 0.2. Consistent with our attentional account, the distractor effect on no-signal trials (i.e., RT distractor minus RT no-distractor) was influenced by the occasional presentation of stop signals in the block: it was smaller in central-signal blocks (58 ms) than in no-signal blocks (70 ms); and was, in turn, smaller in no-signal blocks than in noncentral-signal blocks (83 ms). This interaction between block type and distractor was significant (*p* < .01). Follow-up tests showed that the difference between central and noncentral blocks was significant, *t*(23) = 2.73, *p* = .01 (one-tailed directional *t* test: *p* = .006),^4^ Cohen’s *d* = .55; the differences between central-signal and no-signal blocks, and between no-signal and noncentral blocks were marginally significant; *t*(23) = 1.91, *p* = .07 (one-tailed directional *t* test: *p* = .035), Cohen’s *d* = .39, and *t*(23) = 1.81, *p* = .08 (one-tailed directional *t* test: *p* = .041), Cohen’s *d* = .37, respectively. Collectively, these RT findings support the “attentional” account of proactive stopping, which proposes that ignoring distractors and proactive adjustments in stop-signal tasks both involve (re)focusing visual attention.

## Conclusions

The present study focused on how perception and executive control interact in a stop-signal task. We can draw two main conclusions. First, our results demonstrate that perceptual distractors cause large stopping deficits. This sheds a new light on a range of phenomena and findings in the response inhibition literature. For example, stopping is impaired when incongruent distractors are presented (Chambers et al., 2007; Ridderinkhof, Band, & Logan, 1999; Verbruggen, Liefooghe, & Vandierendonck, 2004). This has been attributed to an interaction between inhibitory processes, but our current results suggest that it could have been caused by adjustments of the attentional focus on distractor trials. Similarly, stopping deficits in certain clinical populations could be due to impairments in selective attention, rather than in inhibition (Bekker et al., 2005). More generally, in everyday life stop signals often occur in noisy environments, so the ability to quickly detect a signal among perceptual distractors may be key to successful stopping. Although some studies have already focused on stop-signal detection (Cavina-Pratesi, Bricolo, Prior, & Marzi, 2001; Jahfari, Ridderinkhof, & Scholte, 2013; van den Wildenberg & van der Molen, 2004), the role of attention and perception has been largely neglected in the response inhibition domain. The dominant view is still to attribute differences in stopping latencies to differences in the effectiveness of a single inhibitory control process (Verbruggen et al., 2014). The present study provides clear behavioral support for the idea that perceptual processes play an important role in reactive stopping. We strongly urge researchers to consider the possibility that intra- or interindividual differences in stopping performance could be caused by differences in stop-signal detection rather than inhibition of motor output when they interpret their findings. Other stop-signal studies have used visual go stimuli and auditory stop signals. This requires divided attention, but we see no reason why stimulus detection would be less important in these situations than in situations in which both signals are presented in the same modality.

Second, the results demonstrate that monitoring for signals is an important aspect of proactive control in the stop-signal task. The relative contribution of various control adjustments, such as signal monitoring, increasing thresholds, or suppressing motor output, may depend on task context. This could explain apparent discrepancies between studies. For example, in Verbruggen and Logan (2009), stop signals were loud auditory signals. We found that both RTs and accuracy on no-signal trials increased in signal blocks, which suggests an increase in response threshold. This idea was supported by diffusion-model fits (Logan, Van Zandt, Verbruggen, & Wagenmakers, 2014; Verbruggen & Logan, 2009). In the present study, we found an increase in RT but a small decrease in accuracy (Tables 1 and 2). This pattern is inconsistent with a response-threshold account. Instead, we propose that the slowing here was mainly caused by monitoring for stop signals. Thus, proactive control in the stop-signal task could be implemented differently, depending on the task context.

The results are consistent with our recently proposed theoretical framework of action control (Verbruggen et al., 2014). We have argued that basic cognitive processes, such as stimulus detection and action selection, underlie most forms of action control, including outright stopping. These processes are, in turn, modulated by processes that operate over a slower time scale, including proactive or preparatory control. The present study highlights the importance of focusing on the underlying processes, such as stimulus detection, rather than general functions, such as response inhibition, as it provides a more precise account of performance.

## Supplementary Material

10.1037/a0036542.supp

## Figures and Tables

**Table 1 tbl1:** Probability of an Accurate Go Response [p(Correct)], Probability of a Missed Go Response [p(Miss)], Average Reaction Time (RT) for Correct Go Responses, Probability of Responding on a Signal Trial [p(Respond)], Average Stop-Signal Delay (SSD), Stop-Signal Reaction Time (SSRT), and Signal-Respond Reaction Time (S-R RT; the Latency of Incorrectly Executed Responses on stop-signal trials) as a Function of Stop-Signal Condition and Distractor Condition

	Central signal				No signal				Noncentral signal			
	No distractor		Distractor		No distractor		Distractor		No distractor		Distractor	
	*M*	*SD*	*M*	*SD*	*M*	*SD*	*M*	*SD*	*M*	*SD*	*M*	*SD*
*p*(correct)	0.95	0.05	0.95	0.05	0.96	0.03	0.96	0.02	0.95	0.05	0.96	0.04
*p*(miss)	0.06	0.05	0.07	0.08	0.02	0.02	0.02	0.03	0.07	0.06	0.08	0.06
RT	946	170	1004	171	719	97	789	97	945	161	1028	156
*p*(respond)	0.47	0.05	0.46	0.05					0.48	0.06	0.56	0.15
SSD	584	204	590	193					555	229	441	280
SSRT	333	92	370	82					363	130	616	260
s-r RT	844	180	902	163					867	151	965	161
*Note.* *P*(Correct) is the ratio of the number of correct responses to the number of correct and incorrect responses: *p*(Correct) = Correct/(Correct + Incorrect). *P*(Miss) is the ratio of the number of omitted responses to the total number of no-stop-signal trials: *p*(Miss) = Missed/(Correct + Incorrect + Missed). *M* = mean; *SD* = standard deviation.												

**Table 2 tbl2:** Overview of Repeated Measures Analyses of Variance Performed to Compare No-Signal and Signal Performance

	*df* 1	*df* 2	SS1	SS2	*F*	*p*	Partial η^2^	Generalized η^2^
Go accuracy								
Signal	2	46	0.004	0.042	1.980	0.150	0.087	0.015
Distract	1	23	0.000	0.010	0.070	0.793	0.000	0.000
Signal:distract	2	46	0.001	0.020	0.957	0.392	0.048	0.004
Go reaction time								
Signal	2	46	1,652,647	763,327	49.796	<0.001	0.684	0.361
Distract	1	23	180,077	9,182	451.053	<0.001	0.951	0.058
Signal:distract	2	46	3,969	16,993	5.372	0.008	0.189	0.001
SSRT								
Signal	1	23	457,826	416,327	25.293	<0.001	0.524	0.167
Distract	1	23	502,189	200,622	57.573	<0.001	0.715	0.180
Signal:distract	1	23	280,899	253,746	25.461	<0.001	0.525	0.109
*Note.* Stop-signal condition (central-signal, noncentral-signal, or no-signal blocks) and distractor (no distractor vs. distractor) are the within-subjects factors. We did not analyze *p*(Miss) because values were low. Note that the main effect of condition for *p*(Correct) was significant when the two outliers were included (*p* = .04). This analysis is presented in the online supplemental material.								

## Footnotes

1 To obtain reliable SSRT estimates with a relatively low number of signal trials, we used a staircase tracking procedure (see the Method section). SSD is continuously adjusted to obtain a probability of stopping of .50. Consequently, we could not use average *p*(respond|signal) as an index of inhibitory control in this study.

2 This study is part of a larger project that focuses on how stopping and both general- and item-specific learning influence decision making in various domains (e.g., Verbruggen, Adams, & Chambers, 2012). To allow comparisons with our other studies, we used a decision-making task in which subjects had to select one of two words that could appear above or below the fixation line.

3 The size of the stimuli and the number of distractors were determined after a pilot study. In this pilot study, we found a small numerical interaction between proactive and reactive stopping and distractor interference, but which failed to reach statistical significance (see online supplemental material). Therefore, in the experiment reported here we sought to increase the effect size by (a) increasing the size of the outer square and, consequently, the distance between the centre of the screen and the noncentral stop signal; (b) increasing the number of distractors; and (c) reducing the width of the stop signals. If perceptual processing is a critical component of reactive and proactive inhibitory control, then increasing the perceptual demands should influence both.

4 The attentional account makes strong predictions about the direction of the distractor effect. No differences in distractor effects or differences in the opposite direction (i.e., the smallest distractor effect in the noncentral condition) would argue against the attentional account of proactive control. Therefore, we report both the two-tailed *p* values and the *p* values of planned one-directional *t* tests.
