# Supplementary material for: Proactive and Reactive Stopping When Distracted: An Attentional Account
Source: J Exp Psychol Hum Percept Perform. 2014 May 19;40(4):1295–300. doi: 10.1037/a0036542 (PMC4120704; doi:10.1037/a0036542)

**Supplemental Materials**

**Proactive and Reactive Stopping When Distracted: An Attentional Account**

**by F. Verbruggen et al., 2014, *Journal of Experimental Psychology: Human Perception and Performance***

**http://dx.doi.org/10.1037/a0036542**

**Pilot study**

Twenty-four students from the University of Exeter participated for monetary compensation (£6). One subject was replaced because their SSRT was 6 standard deviations above the average in the non-central distractor condition. Inclusion of this subject did not alter the results in a meaningful way. The procedure was similar to the one used in the main experiment reported in the manuscript, except for a few differences: the size of the outer square was smaller (7.5°x°7.5), the stop signals were wider (5 pixels), the number of distractors was lower (15), and the center of the distractors was outside a smaller central region (2.1°x°2.1).

Relevant no-signal and signal data appear in Tables S1–2, and Table S3 provides an overview of the repeated Analyses of Variance performed to compare no-signal and signal performance.

Subjects required more time to stop their response on distractor trials (371 ms) than on no-distractor trials (272 ms), *p* < .05 (Table S3). This distractor effect was numerically larger in the non-central signal condition (113 ms) than in the central signal condition (85 ms), but this difference was not significant (*p* = .18; Table S3). The main effect of signal was also not significant.

Average RT of correct no-signal trials was longer in the central (921 ms) and non-central (906 ms) signal blocks than in no-signal blocks (768 ms). The presentation of distractors slowed responses (no-distractor trials: 836 ms; distractor trials: 893 ms). Both main effects were significant (p < .001; Table S3). Distractors had a numerically larger effect in non-central signal blocks (64 ms) than in no-signal (55 ms) blocks, but the distractor effect was similar in no-signal and central-signal blocks (52 ms), and the overall interaction between distractor and block type was not significant (*p* = .23).

In summary, we found some differences between signal conditions, but the crucial interactions between block type and distractor presentation did not reach significance. Therefore, in the experiment reported in the main manuscript we (a) increased the size of the outer square, and consequently, the distance between the center of the screen and the non-central stop signal; (b) increased the number of distractors; and (c) reduced the width of the stop signals. By increasing the perceptual demands, we expected more pronounced interactions between perceptual–interference control and reactive and proactive control.

**Additional behavioural analyses main experiment**

In the experiment reported in the manuscript, 2 subjects were replaced because the percentage of correct go trials was ≤ 75%. In Tables S1 and S2, we show the group averages for all conditions when these subjects were included in the analyses. We also reran all analyses (Table S3). Exclusion of these subjects did not alter the results substantially. All main effects and interactions were significant for no-signal RT and SSRT. This is consistent with the results reported in the main text. The Go Accuracy analyses now revealed a significant main effect of signal block, which suggests that accuracy was lower in the signal blocks than in the no-signal blocks (see also Table S1).

Table S1

*Probability of an Accurate Go Response [p(correct)], Probability of a Missed Go Response [p(miss)], and Average Reaction Time for Correct Go Responses, as a Function of Experiment, Stop-Signal Condition, and Distractor Condition*

|  |  | | *p(correct)* | | *p(miss)* | | *Reaction time* | |
| --- | --- | --- | --- | --- | --- | --- | --- | --- |
|  |  | | *M* | *sd* | *M* | *sd* | *M* | *sd* |
| Pilot study | | |  |  |  |  |  |  |
|  | Central signal | |  |  |  |  |  |  |
|  |  | No distractor | 0.96 | 0.03 | 0.04 | 0.04 | 894 | 168 |
|  |  | Distractor | 0.96 | 0.04 | 0.05 | 0.04 | 947 | 164 |
|  | Non-central signal | |  |  |  |  |  |  |
|  |  | No distractor | 0.96 | 0.04 | 0.04 | 0.03 | 874 | 149 |
|  |  | Distractor | 0.96 | 0.04 | 0.04 | 0.03 | 938 | 145 |
|  | No signal | |  |  |  |  |  |  |
|  |  | No distractor | 0.95 | 0.04 | 0.02 | 0.02 | 740 | 75 |
|  |  | Distractor | 0.96 | 0.03 | 0.02 | 0.03 | 795 | 80 |
| Main Experiment (*N* = 26) | | |  |  |  |  |  |  |
|  | Central signal | |  |  |  |  |  |  |
|  |  | No distractor | 0.946 | 0.05 | 0.07 | 0.06 | 955 | 173 |
|  |  | Distractor | 0.943 | 0.05 | 0.08 | 0.08 | 1014 | 172 |
|  | Non-central signal | |  |  |  |  |  |  |
|  |  | No distractor | 0.942 | 0.05 | 0.08 | 0.07 | 956 | 161 |
|  |  | Distractor | 0.949 | 0.04 | 0.10 | 0.09 | 1039 | 159 |
|  | No signal | |  |  |  |  |  |  |
|  |  | No distractor | 0.962 | 0.03 | 0.03 | 0.03 | 730 | 101 |
|  |  | Distractor | 0.958 | 0.03 | 0.03 | 0.03 | 803 | 105 |

*Note*. In the main experiment, all subjects (including outliers) were included. *P*(correct) is the ratio of the number of correct responses to the number of correct and incorrect responses: *p*(correct) = correct/(correct + incorrect). *P*(miss) is the ratio of the number of omitted responses to the total number of no-stop-signal trials: *p*(miss) = missed/(correct + incorrect + missed). M = mean; sd = standard deviation; N = total number of subjects.

Table S2

*Probability of Responding on a Signal Trial [p(respond)], Average Stop-Signal Delay (SSD), Stop-Signal Reaction Time (SSRT), and Signal-Respond Reaction Time (i.e. Latency of Incorrectly Executed Responses) as a Function of Experiment, Stop-Signal Condition, and Distractor Condition*.

|  |  | | *p(respond)* | | *SSD* | | *SSRT* | | *Signal-respond RT* | |
| --- | --- | --- | --- | --- | --- | --- | --- | --- | --- | --- |
|  |  | | *M* | *sd* | *M* | *sd* | *M* | *sd* | *M* | *sd* |
| Pilot study | | |  |  |  |  |  |  |  |  |
|  | Central signal | |  |  |  |  |  |  |  |  |
|  |  | No distractor | 0.48 | 0.05 | 591 | 185 | 273 | 58 | 778 | 155 |
|  |  | Distractor | 0.48 | 0.05 | 560 | 163 | 358 | 65 | 829 | 140 |
|  | Non-central signal | |  |  |  |  |  |  |  |  |
|  |  | No distractor | 0.47 | 0.05 | 571 | 185 | 270 | 79 | 757 | 140 |
|  |  | Distractor | 0.48 | 0.06 | 529 | 189 | 383 | 81 | 829 | 124 |
| Main Experiment (*N* = 26) | | |  |  |  |  |  |  |  |  |
|  | Central signal | |  |  |  |  |  |  |  |  |
|  |  | No distractor | 0.47 | 0.05 | 588 | 202 | 337 | 89 | 853 | 178 |
|  |  | Distractor | 0.46 | 0.05 | 596 | 195 | 376 | 83 | 916 | 170 |
|  | Non-central signal | |  |  |  |  |  |  |  |  |
|  |  | No distractor | 0.48 | 0.06 | 588 | 202 | 337 | 89 | 876 | 152 |
|  |  | Distractor | 0.55 | 0.14 | 596 | 195 | 376 | 83 | 978 | 164 |

*Note*. In the main experiment, all subjects (including outliers) were included. M = mean; sd = standard deviation; *N* = number of subjects.

Table S3

*Overview of Repeated Measures Analyses of Variance Performed to Compare No-Signal and Signal Performance in the Pilot Study and the Main Experiment When All Subjects Were Included*

|  |  | | *df 1* | *df2* | *SS1* | *SS2* | *F* | *p* | *partial η^2^* | *gen. η^2^* |
| --- | --- | --- | --- | --- | --- | --- | --- | --- | --- | --- |
| Pilot study | | |  |  |  |  |  |  |  |  |
|  | Go accuracy | |  |  |  |  |  |  |  |  |
|  |  | Signal | 2 | 46 | 0.001 | 0.017 | 0.729 | 0.488 | 0.056 | 0.003 |
|  |  | Distract | 1 | 23 | 0.000 | 0.010 | 0.726 | 0.403 | 0.000 | 0.002 |
|  |  | Signal:distract | 2 | 46 | 0.001 | 0.013 | 2.046 | 0.141 | 0.071 | 0.006 |
|  | Go Reaction Time | |  |  |  |  |  |  |  |  |
|  |  | Signal | 2 | 46 | 683878 | 410529 | 38.314 | 0.000 | 0.625 | 0.212 |
|  |  | Distract | 1 | 23 | 116204 | 14700 | 181.812 | 0.000 | 0.888 | 0.044 |
|  |  | Signal:distract | 2 | 46 | 894 | 13705 | 1.500 | 0.234 | 0.061 | 0.000 |
|  | SSRT | |  |  |  |  |  |  |  |  |
|  |  | Signal | 1 | 23 | 2884 | 86336 | 0.768 | 0.390 | 0.032 | 0.006 |
|  |  | Distract | 1 | 23 | 235859 | 43490 | 124.735 | 0.000 | 0.844 | 0.334 |
|  |  | Signal:distract | 1 | 23 | 4330 | 53409 | 1.865 | 0.185 | 0.075 | 0.009 |
| Main experiment (*N* = 26) | | |  |  |  |  |  |  |  |  |
|  | Go accuracy | |  |  |  |  |  |  |  |  |
|  |  | Signal | 2 | 50 | 0.007 | 0.054 | 3.438 | 0.040 | 0.115 | 0.025 |
|  |  | Distract | 1 | 25 | 0.000 | 0.012 | 0.003 | 0.954 | 0.000 | 0.000 |
|  |  | Signal:distract | 2 | 50 | 0.001 | 0.024 | 1.010 | 0.372 | 0.040 | 0.003 |
|  | Go Reaction Time | |  |  |  |  |  |  |  |  |
|  |  | Signal | 2 | 50 | 1758393 | 791673 | 55.528 | 0.000 | 0.690 | 0.348 |
|  |  | Distract | 1 | 25 | 199240 | 9451 | 527.021 | 0.000 | 0.955 | 0.057 |
|  |  | Signal:distract | 2 | 50 | 3917 | 19970 | 4.903 | 0.011 | 0.164 | 0.001 |
|  | SSRT | |  |  |  |  |  |  |  |  |
|  |  | Signal | 1 | 25 | 481344 | 421757 | 28.530 | 0.000 | 0.929 | 0.172 |
|  |  | Distract | 1 | 25 | 555130 | 202043 | 68.690 | 0.000 | 0.533 | 0.193 |
|  |  | Signal:distract | 1 | 25 | 299714 | 254294 | 29.470 | 0.000 | 0.733 | 0.115 |

*Note*. Stop-signal condition (central-signal, non-central signal, or no-signal blocks) and distractor (no distractor vs. distractor) are the within-subjects factors. We did not analyse p(miss) because values were low. *gen. η^2^* = generalised eta squared.

**Go stimuli**

We used the 54 words in this study.

Natural:

gull, pear, wasp, moth, calf, plum, crow, slug, leaf, dove, toad, swan, crab, pony, deer, worm, lamb, goat, frog, hawk, rice, lion, wolf, duck, bull, bear, tree.

Man-made:

tuba, tram, coil, mast, tile, gong, harp, wand, vase, raft, sofa, drum, fork, sock, coin, jeep, shed, pill, barn, sink, flag, pipe, bowl, belt, shoe, desk, book.

**Analyses of the eye data**

An EyeLink 1000 Desktop Mount camera system (SR Research, Ottawa, Canada), calibrated before each block, tracked the gaze position of the right eye during the whole block (sampling rate: 500 Hz). The EyeLink was calibrated and controlled via Psychtoolbox (Cornelissen, Peters, & Palmer, 2002). Eye data were subsequently exported using the Eyelink Data Viewer (SR Research, Ottawa, Canada): for each subject, we generated a file with information about all fixations and a file with trial information and the sequence of events. We integrated these files using R, and created a large data file for further analyses.

In the analyses of the eye data, we excluded subjects when no fixation was registered at the beginning of an event (e.g. presentation of the go stimulus) on more than 15% of the trials, as this could indicate that eye-movement registration was suboptimal. Based on this criterion, we excluded 5 subjects in the pilot and 6 subjects in the main experiment. Note that inclusion of those subjects did not alter the results much (not shown). We also excluded all fixations that were supposedly off screen (0.2% in the pilot and 0.5% in the main experiment).

In the analyses, we focused on the number of the fixations and the fixation location for three intervals: (1) the fixation interval, (2) the interval between the presentation of the go stimulus and the response on no-signal trials, and (3) after the stop signal. In the pilot, eye movements made 400 ms after the presentation of the stop signal were excluded; in the main experiment, we used 700 ms as a cut-off. These values were based on the largest average SSRT value (in both the pilot study and the main experiment, this was SSRT in the non-central distractor condition).

The number of fixations and fixation location for each trial type (distractor vs. no-distractor) and block type (central-signal, non-central signal, and no-signal) could provide further information on how subjects controlled perceptual interference and monitored for the occurrence of occasional stop signals. For example, people may narrow attention to reduce distractor processing; potentially, this could also reduce the number of fixations towards distractors. Similarly, monitoring for central stop signals could lead to more fixations on central locations in central-signal blocks than in no-signal blocks. By contrast, detecting non-central stop signals requires a wider attentional focus; and this could result in more fixations towards the non-central square in non-central signal blocks than in the two other block types.

The descriptive statistics are in Tables S4 (pilot) and S6 (main experiment); the inferential statistics are in Tables S5 (pilot) and S7 (main experiment). If the number of fixations for a particular interval = 1, then the subject did not move their eyes during the whole interval (i.e. the eyes remained fixated on the region that was fixated before the interval had started). Fixation location can be analysed in different ways. Here we calculated the average distance between the fixated location and the center of the screen [distance = sqrt(x-coordinate^2 + y-coordinate^2)]. In Tables S4 and S6, we also show the proportion of the fixations that fell within 4 pre-defined regions: a region in the center of the screen (around the stop-signal and the two words), a region in which the distractors occurred, a region around the non-central stop-signal, and an outside region; these regions with their coordinates are depicted in Figure S1.

As can be seen in the tables, subjects did not make many eye movements during the intervals of interest, and they fixated on the center of the screen most of the time. In the pilot study, the numerical differences between signal and distractor conditions were very small. All p values ≥ .05, except for the effect of signal type on the number of fixations on signal trials: subjects tended to make more eye movements in non-central signal trials than in central-signal trials, *p* = .015.

In the main experiment, the differences between signal conditions were slightly larger (Table S6). The average distance between fixated location and the central location was larger in non-central blocks than in central-signal and no-signal blocks; they also made more eye movements in the non-central blocks (see highlighted cells in Table S6). These differences were reliable (*p*’s < .03; Table S7). This is consistent with the idea that subjects widened attention in the non-central signal condition. On stop-signal trials, subjects also made more eye movements towards the outer square in non-central signal blocks than in central-signal blocks (Tables S6-7; *p*’s < .04). In other words, when stop signals in the periphery were harder to detect, subjects were more likely to move their eyes to the location of the stop signal. Despite differences being small, the results of the main experiment are largely consistent with the ‘attentional’ account.

**Reference:**

Cornelissen, F. W., Peters, E. M., & Palmer, J. (2002). The eyelink toolbox: Eye tracking with MATLAB and the psychophysics toolbox. *Behaviour Research Methods, 34*, 613–617.

Table S4

*An Overview of the Number of Fixations, Average Distance Between Fixation Location and Center of the Screen (in Pixels), and the Proportion of the Fixations That Fell Within Our 4 Pre-defined Regions, Per Event (Fixation Interval, Go Stimulus-Response Interval on No-Signal Trials, and After the Presentation of the Stop Signal) and for Each Signal and Distractor Condition in the Pilot Study*

|  | | Number of Fixations | | Distance | | Probability  central region | | Probability  distractor region | | Probability  NCS  region | | Probability  outside  region | |
| --- | --- | --- | --- | --- | --- | --- | --- | --- | --- | --- | --- | --- | --- |
|  | | *M* | *sd* | *M* | *sd* | *M* | *sd* | *M* | *sd* | *M* | *sd* | *M* | *sd* |
| Fixation interval | |  |  |  |  |  |  |  |  |  |  |  |  |
|  | Central | 1.09 | 0.06 | 31.13 | 9.33 | 0.73 | 0.20 | 0.27 | 0.20 | 0.00 | 0.00 | 0.00 | 0.00 |
|  | Non-central | 1.09 | 0.06 | 31.60 | 11.21 | 0.71 | 0.22 | 0.28 | 0.22 | 0.00 | 0.00 | 0.01 | 0.01 |
|  | No-signal | 1.08 | 0.07 | 34.75 | 12.61 | 0.67 | 0.26 | 0.32 | 0.26 | 0.00 | 0.01 | 0.01 | 0.01 |
| Go stimulus | |  |  |  |  |  |  |  |  |  |  |  |  |
|  | Central,  no distractor | 1.57 | 0.30 | 31.33 | 9.98 | 0.71 | 0.20 | 0.29 | 0.20 | 0.00 | 0.00 | 0.00 | 0.00 |
|  | Central,  distractor | 1.54 | 0.23 | 30.29 | 9.47 | 0.73 | 0.19 | 0.27 | 0.19 | 0.00 | 0.00 | 0.00 | 0.00 |
|  | Non-central,  no distractor | 1.63 | 0.32 | 31.33 | 10.87 | 0.70 | 0.23 | 0.30 | 0.23 | 0.00 | 0.00 | 0.00 | 0.01 |
|  | Non-central,  distractor | 1.62 | 0.28 | 31.31 | 10.60 | 0.71 | 0.21 | 0.29 | 0.21 | 0.00 | 0.00 | 0.00 | 0.01 |
|  | No-signal,  no distractor | 1.55 | 0.21 | 35.08 | 13.17 | 0.65 | 0.25 | 0.34 | 0.25 | 0.00 | 0.00 | 0.01 | 0.01 |
|  | No-signal,  distractor | 1.54 | 0.20 | 34.47 | 12.61 | 0.67 | 0.25 | 0.32 | 0.25 | 0.00 | 0.00 | 0.00 | 0.01 |
| Stop signal | |  |  |  |  |  |  |  |  |  |  |  |  |
|  | Central,  no distractor | 1.18 | 0.09 | 30.74 | 10.05 | 0.72 | 0.21 | 0.27 | 0.21 | 0.00 | 0.00 | 0.00 | 0.01 |
|  | Central,  distractor | 1.17 | 0.08 | 29.59 | 8.92 | 0.75 | 0.19 | 0.25 | 0.19 | 0.00 | 0.00 | 0.00 | 0.00 |
|  | Non-central,  no distractor | 1.23 | 0.10 | 30.86 | 10.34 | 0.71 | 0.22 | 0.29 | 0.22 | 0.00 | 0.00 | 0.00 | 0.00 |
|  | Non-central,  distractor | 1.21 | 0.13 | 30.45 | 9.31 | 0.72 | 0.21 | 0.28 | 0.21 | 0.00 | 0.00 | 0.00 | 0.01 |

*Note*. NCS = non-central stop-signal region; M = mean; sd = standard deviation.

Table S5

*Overview of Analyses of Variance Performed to Compare Number of Fixations and Average Distance in the Pilot Study*

|  |  | | *df 1* | *df2* | *SS1* | *SS2* | *F* | *p* | *partial η^2^* | *gen. η^2^* |
| --- | --- | --- | --- | --- | --- | --- | --- | --- | --- | --- |
| Fixation interval | | |  |  |  |  |  |  |  |  |
|  | Number of fixations | |  |  |  |  |  |  |  |  |
|  |  | Signal | 2 | 36 | 0.003 | 0.025 | 1.833 | 0.175 | 0.107 | 0.012 |
|  | Distance | |  |  |  |  |  |  |  |  |
|  |  | Signal condition | 2 | 36 | 146.549 | 1255.236 | 2.102 | 0.137 | 0.105 | 0.021 |
| Go stimuli | | |  |  |  |  |  |  |  |  |
|  | Number of fixations | |  |  |  |  |  |  |  |  |
|  |  | Signal | 2 | 36 | 0.124 | 0.683 | 3.257 | 0.050 | 0.154 | 0.016 |
|  |  | Distract | 1 | 18 | 0.009 | 0.216 | 0.787 | 0.387 | 0.040 | 0.001 |
|  |  | Signal:distract | 2 | 36 | 0.002 | 0.132 | 0.234 | 0.793 | 0.015 | 0.000 |
|  | Distance | |  |  |  |  |  |  |  |  |
|  |  | Signal condition | 2 | 36 | 352.717 | 2175.221 | 2.919 | 0.067 | 0.140 | 0.025 |
|  |  | Distract | 1 | 18 | 8.848 | 44.397 | 3.587 | 0.074 | 0.166 | 0.001 |
|  |  | Signal:distract | 2 | 36 | 5.040 | 60.837 | 1.491 | 0.239 | 0.077 | 0.000 |
| Stop signal | | |  |  |  |  |  |  |  |  |
|  | Number of fixations | |  |  |  |  |  |  |  |  |
|  |  | Signal | 1 | 18 | 0.036 | 0.090 | 7.272 | 0.015 | 0.286 | 0.046 |
|  |  | Distract | 1 | 18 | 0.004 | 0.047 | 1.438 | 0.246 | 0.078 | 0.005 |
|  |  | Signal:distract | 1 | 18 | 0.000 | 0.059 | 0.013 | 0.909 | 0.000 | 0.000 |
|  | Distance | |  |  |  |  |  |  |  |  |
|  |  | Signal | 1 | 18 | 4.552 | 698.701 | 0.117 | 0.736 | 0.006 | 0.001 |
|  |  | Distract | 1 | 18 | 11.629 | 60.573 | 3.456 | 0.079 | 0.161 | 0.002 |
|  |  | Signal:distract | 1 | 18 | 2.639 | 83.037 | 0.572 | 0.459 | 0.031 | 0.000 |

Table S6

*An Overview of the Number of Fixations, Average Distance Between Fixation Location and Center of the Screen (in Pixels), and the Proportion of the Fixations That Fell Within our 4 Predefined Regions, Per Event (Fixation Interval, Go Stimulus-Response Interval on No-Signal Trials, and After the Presentation of the Stop Signal) and for Each Signal and Distractor Condition in the Main Experiment*

|  | | Number of Fixations | | Distance | | Probability  central region | | Probability  distractor region | | Probability  NCS  region | | Probability  outside  region | |
| --- | --- | --- | --- | --- | --- | --- | --- | --- | --- | --- | --- | --- | --- |
|  | | *M* | *sd* | *M* | *sd* | *M* | *sd* | *M* | *sd* | *M* | *sd* | *M* | *sd* |
| Fixation interval | |  |  |  |  |  |  |  |  |  |  |  |  |
|  | Central | 1.13 | 0.10 | 36.03 | 11.49 | 0.67 | 0.23 | 0.31 | 0.23 | 0.00 | 0.01 | 0.01 | 0.01 |
|  | Non-central | 1.18 | 0.14 | 35.99 | 10.79 | 0.68 | 0.20 | 0.31 | 0.19 | 0.00 | 0.00 | 0.01 | 0.01 |
|  | No-signal | 1.08 | 0.06 | 37.22 | 15.93 | 0.66 | 0.28 | 0.33 | 0.28 | 0.00 | 0.00 | 0.01 | 0.02 |
| Go stimulus | |  |  |  |  |  |  |  |  |  |  |  |  |
|  | Central,  no distractor | **1.40** | 0.32 | **38.84** | 16.94 | 0.66 | 0.24 | 0.32 | 0.23 | 0.00 | 0.01 | 0.02 | 0.04 |
|  | Central,  distractor | **1.39** | 0.34 | **36.95** | 15.96 | 0.68 | 0.23 | 0.30 | 0.22 | 0.00 | 0.01 | 0.02 | 0.04 |
|  | Non-central,  no distractor | **1.80** | 0.62 | **50.03** | 26.07 | 0.61 | 0.23 | 0.34 | 0.20 | 0.02 | 0.03 | 0.02 | 0.05 |
|  | Non-central,  distractor | **1.75** | 0.60 | **51.59** | 30.28 | 0.61 | 0.25 | 0.33 | 0.21 | 0.02 | 0.03 | 0.03 | 0.07 |
|  | No-signal,  no distractor | **1.42** | 0.22 | **37.58** | 15.57 | 0.64 | 0.27 | 0.35 | 0.27 | 0.00 | 0.00 | 0.01 | 0.02 |
|  | No-signal,  distractor | **1.40** | 0.26 | **37.44** | 15.61 | 0.65 | 0.26 | 0.34 | 0.26 | 0.00 | 0.00 | 0.01 | 0.02 |
| Stop signal | |  |  |  |  |  |  |  |  |  |  |  |  |
|  | Central,  no distractor | **1.27** | 0.15 | **37.77** | 12.82 | 0.65 | 0.24 | 0.34 | 0.24 | 0.01 | 0.02 | 0.01 | 0.03 |
|  | Central,  distractor | **1.24** | 0.18 | **34.91** | 12.19 | 0.68 | 0.24 | 0.31 | 0.24 | 0.00 | 0.01 | 0.01 | 0.03 |
|  | Non-central,  no distractor | **1.45** | 0.32 | **51.88** | 35.78 | 0.61 | 0.26 | 0.33 | 0.21 | 0.03 | 0.05 | 0.03 | 0.09 |
|  | Non-central,  distractor | **1.41** | 0.30 | **54.37** | 35.74 | 0.60 | 0.26 | 0.33 | 0.23 | 0.03 | 0.04 | 0.04 | 0.10 |

*Note*. NCS = non-central stop-signal region; M = mean; sd = standard deviation. Contrasts discussed in text are in bold.

Table S7

*Overview of Analyses of Variance Performed to Compare Number of Fixations and Average Distance in the Main Experiment*

|  |  | | *df 1* | *df2* | *SS1* | *SS2* | *F* | *p* | *partial η^2^* | *gen. η^2^* |
| --- | --- | --- | --- | --- | --- | --- | --- | --- | --- | --- |
| Fixation interval | | |  |  |  |  |  |  |  |  |
|  | Number of fixations | |  |  |  |  |  |  |  |  |
|  |  | Signal condition | 2 | 36 | 0.090 | 0.139 | 11.649 | 0.000 | 0.393 | 0.127 |
|  | Distance | |  |  |  |  |  |  |  |  |
|  |  | Signal condition | 2 | 36 | 18.644 | 1423.725 | 0.236 | 0.791 | 0.013 | 0.002 |
| Go stimuli | | |  |  |  |  |  |  |  |  |
|  | Number of fixations | |  |  |  |  |  |  |  |  |
|  |  | Signal | **2** | **36** | **3.478** | **7.434** | **8.421** | **0.001** | **0.319** | **0.153** |
|  |  | Distract | 1 | 18 | 0.023 | 0.232 | 1.809 | 0.195 | 0.090 | 0.001 |
|  |  | Signal:distract | 2 | 36 | 0.008 | 0.223 | 0.671 | 0.517 | 0.035 | 0.000 |
|  | Distance | |  |  |  |  |  |  |  |  |
|  |  | Signal | **2** | **36** | **4358.285** | **19593.499** | **4.004** | **0.027** | **0.182** | **0.084** |
|  |  | Distract | 1 | 18 | 0.735 | 233.029 | 0.057 | 0.814 | 0.003 | 0.000 |
|  |  | Signal:distract | 2 | 36 | 56.643 | 469.107 | 2.173 | 0.128 | 0.108 | 0.001 |
| Stop signal | | |  |  |  |  |  |  |  |  |
|  | Number of fixations | |  |  |  |  |  |  |  |  |
|  |  | Signal | **1** | **18** | **0.607** | **0.828** | **13.202** | **0.002** | **0.423** | **0.119** |
|  |  | Distract | 1 | 18 | 0.020 | 0.144 | 2.447 | 0.135 | 0.122 | 0.004 |
|  |  | Signal:distract | 1 | 18 | 0.000 | 0.064 | 0.052 | 0.822 | 0.000 | 0.000 |
|  | Distance | |  |  |  |  |  |  |  |  |
|  |  | Signal | **1** | **18** | **5353.255** | **19502.093** | **4.941** | **0.039** | **0.215** | **0.094** |
|  |  | Distract | 1 | 18 | 0.670 | 216.162 | 0.056 | 0.816 | 0.003 | 0.000 |
|  |  | Signal:distract | 1 | 18 | 136.062 | 485.052 | 5.049 | 0.037 | 0.219 | 0.003 |

*Note*. Contrasts discussed in text are in bold.

*Figure S1*. To analyse fixation location, we predefined 4 regions (squares): a central region around the central stop signal and the two words, a region with the distractors, a region around the non-central stop-signal, and an outside region. Size of each square is in pixels because pixel coordinates were used for registration of fixation location (values pilot between brackets). See main text for visual angles. Screen size: 1024 x 768 pixels.


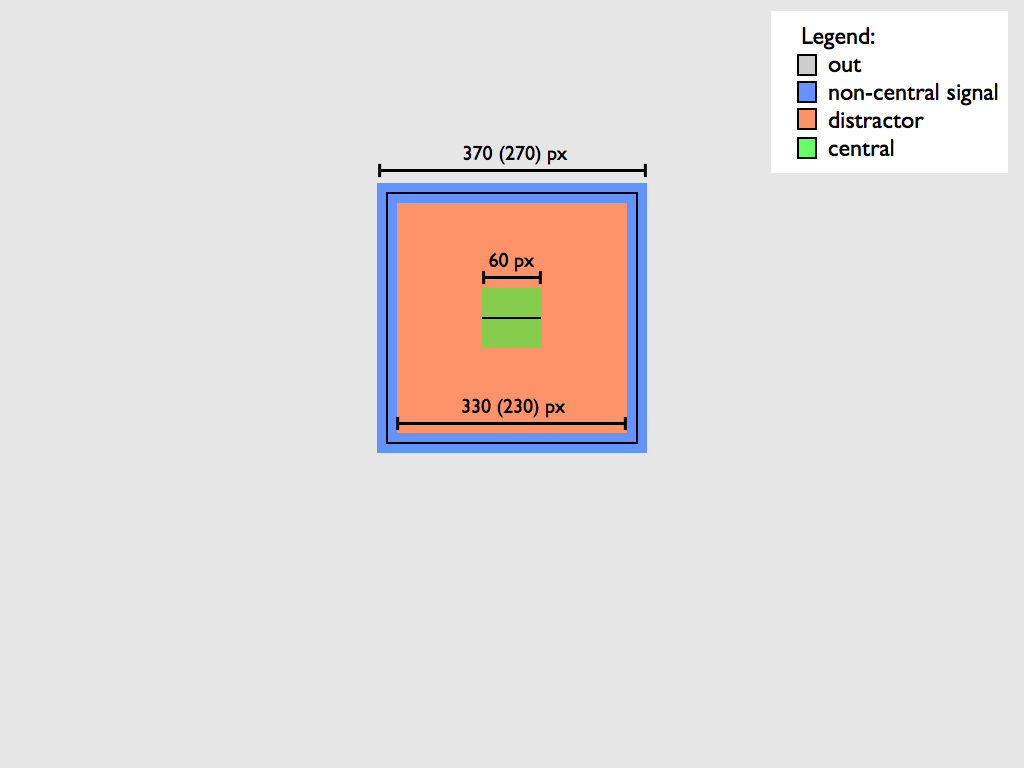

Supplement: Supplementary file 1 [file zfn003143077s1.final.doc]
